# Development of Biomimetic NiTi Alloy: Influence of Thermo-Chemical Treatment on the Physical, Mechanical and Biological Behavior

**DOI:** 10.3390/ma9060402

**Published:** 2016-05-24

**Authors:** Elisa Rupérez, José María Manero, Luis-Alberto Bravo-González, Eduardo Espinar, F.J. Gil

**Affiliations:** 1 Centre de Recerca Nanoenginyeria (CrnE), Departamento Ciencia de los Materiales e Ingeniería Metalúrgica, Escola Tècnica Superior d’Enginyeria Industrial de Barcelona (ETSEIB), Universidad Politécnica de Catalunya, Barcelona 08028, Spain; jose.maria.manero@upc.edu; 2 Unidad Docente de Ortodoncia, Facultad de Odontología, Universidad de Murcia, Murcia 30003, Spain; bravo@um.es; 3 Department of Ortodoncia, Facultad de Odontología, Universidad de Sevilla, Sevilla 41009, Spain; eespinar@us.es; 4 Universidad Internacional de Cataluña, C/Immaculada 22, Barcelona 08195, Spain; xavier.gil@uic.cat

**Keywords:** bioactivity, NiTi alloy, superelasticity, shape memory effect, biomimetic surfaces

## Abstract

A bioactive layer, free of nickel, has been performed for its greater acceptability and reliability in clinical applications for NiTi shape memory alloys. In the first step, a safe barrier against Ni release has been produced on the surface by means of a thicker rutile/anastase protective layer free of nickel. In the second step, a sodium alkaline titanate hydrogel, which has the ability to induce apatite formation, has been performed from oxidized surface. An improvement of host tissue–implant integration has been achieved in terms of Ni ions release and the bioactivity of the treated NiTi alloys has been corroborated with both *in vitro* and *in vivo* studies. The transformation temperatures (A_s_, A_f_, M_s_, and M_f_), as well as the critical stresses (σ^β⇔M^), have been slightly changed due to this surface modification. Consequently, this fact must be taken into account in order to design new surface modification on NiTi implants.

## 1. Introduction

NiTi shape memory alloy (NiTi SMA) is an interesting material in the biomedical area due to its unusual properties such us one-way and two-way shape memory effects, superelastic effect, high damping property and rubber-like effect [1]. For these reasons, NiTi SMA has been used for different orthopedic applications such as load-bearings plates for bone fracture repair, internal fixations for long bone shafts, spinal correctors, vertebral spacers or bone distraction devices [2,3]. However, for long-term medical outcomes, some concerns still remain to be addressed; for example, its potential toxicity and bioactivity [4,5,6].

Concerning toxicity, it is well reported that nickel ions release from the surface (Ni dissolution or wear debries) may cause some allergic reaction and biocompatibility problems [4,5,6]. The protective nickel-titanium oxide layer acts as a barrier to chemical attack and corrosion and confines the diffusion of Ni ions, however, this oxide layer can be depleted by wear, corrosion and fatigue. By means of surface oxidation (laser or plasma source ion implantation techniques), it is possible to reduce the amount of Ni ions leaching from the surface of NiTi implant to negligible amounts [7,8]. Nevertheless, one of the major issues has proven to be an inadequate interaction between the NiTi SMA and the surrounding natural tissue leading to foreign body reactions [9]. For example, the appearance of a dense layer of fibrotic connective tissue at interface of bone–implant could generate weak anchorage between the implant and adjacent bone leading to micro-motion and, finally, loosening of the implant. Therefore, preparation of a bioactive layer, free of nickel, is regarded as a critical issue for its greater acceptability and reliability in clinical applications.

Development of calcium phosphate layers on implants surface are considered to be one of the prospective methods for improving bioactivity [10,11,12]. Different surface techniques, for example, thermal spray, physical vapor deposition, electrodeposition or sol-gel techniques, have been employed to provide metal implants with bone-bonding ability [13,14,15]. Mediaswanti *et al.* [16] presented a summary of the characteristics of the various coating techniques for calcium phosphate showing its own benefits and drawbacks respectively.

Recently, it has been reported that the development of biomimetic surfaces by thermochemical treatments is a promising method due to its ability to produce coating complex shapes with tunables chemical composition of the coating [16]. Some time ago, Kokubo [17] demonstrated that an *in vitro* chemical-deposited bonelike apatite on cp Ti could be induced by an alkali and heat treatment process followed by a simulated body fluid (SBF) soaking. This apatite layer does not have the problems associated with the apatite produced by plasma-spray technique; for example, delamination or decomposition at high temperatue [16]. For NiTi SMA, there are few studies about development of biomimetic surfaces by thermochemical treatments to improve host tissue–implant integration [18,19]. For example, Chen *et al.* reported an apatite layer deposited on NiTi by treating the alloy with HNO_3_ and then NaOH aqueous solution in SBF [19]. They demonstrated that osteoblasts actively proliferated on the HA coating at six weeks after implantation.

In the present study, a modified thermo-chemical treatment established by Kokubo for inducing bioactivity of nickel-titanium has been evaluated. The first step was focused on the surface oxidation in order to decrease the Ni ion concentration, whereas the second step was a thermochemical treatment with NaOH aqueous solution for inducing bioactivity. Finally, the effect of the superelastic/shape memory properties in terms of transformation temperatures and critical stress transformations has been evaluated. In terms of clinical applications, the variation of the critical stresses implies that higher or lower forces will be applied to the bone tissues. Therefore, for development of NiTi biomimetic surfaces, these considerations must be taken into account.

## 2. Materials and Methods

TiNi samples taken from commercial alloys (Memory Metalle GmbH, Weil am Rhein, Germany) designed for shape memory applications with martensite phase (Alloy M: 48.5 at % Ni, wire, hot rolled) and superelasticity with austenite phase (Alloy A: 51.5 at % Ni, thin plate, annealed) were selected as object of the study. Samples were treated at 400 °C during 2.5 h in a low-oxygen pressure atmosphere (3 × 10^−2^ mbar) [20]. Afterward, they were treated with 0.5 M NaOH aqueous solution at 60 °C for 24 h, washed gently with distilled water and dried at 40 °C for 24 h. Subsequently, they were heated up to 600 °C at a rate of 5 °C/min in an electric furnace, kept at 600 °C for 1 h and then allowed to cool in the furnace [17]. Finally, the treated nickel-titanium substrates were soaked in an acellular simulated body fluid (SBF). It is a solution with pH = 7.40 and an ion concentration nearly equal to those of human blood plasma [21]. Each sample was soaked in 40 mL of SBF at 37 °C for 2 weeks. The SBF was renewed every two days.

The crystallographic studies were analyzed by Grazing-Incidence X-ray Diffraction (GI-XRD) in an automatic diffractometer (X’Pert MPD, Philips, Eindhoven, The Netherlands) equipped with a thin film attachment, using Cu-Kα radiation. A range of 2θ = 20°–50° was studied in continuous mode, under a fixed incidence angle of θ_f_ = 0.5°.

The chemical composition of the surfaces was analyzed by X-ray photoelectron spectroscopy (XPS) (SPECS Surface Nano Analysis GmbH, Berlin, Germany). Measurements were performed using an XR50 Mg anode source operating at 150 W and a Phoibos 150 MCD-9 detector (D8 advance, SPECS Surface Nano Analysis GmbH, Berlin, Germany). High resolution spectra were collected using 25 eV at 0.1 eV steps with a chamber pressure below 7.5 × 10^−9^ mbar. Binding energies were calibrated using the C 1s signal at 480 eV. The XPS depth profiles were recorded by using a rastered 3 keV Ar+ ion beam. The etching rate is about 2.3 nm/min. Two specimens were analyzed for each studied condition.

The transformation temperatures (M_s_, M_f_, A_s_ A_f_) of the alloy obtained were determined by Differential Scanning Calorimetry (DSC, 2920 modulated DSC, TA Instruments, New Castle, DE, USA) with a heating ramp of 10 °C/min and a cooling ramp of 2 °C/min.

Tensile tests were carried out on a servo-hydraulic testing machine (MTS-Bionix 858, Eden Prairie, MN, USA), working at a cross-head speed of 10 mm/min. Five NiTi specimens tested were cylinders of 0.457 mm in diameter and of 45 mm in length. The critical stresses (austenite to stress-induced martensite) were determined from these tests, which were performed in simulated body fluid at 37 °C.

For the nickel ion release, samples were immersed in 6 mL of conventional SBF at pH = 7.4, 37 °C until 30 days. At Day 5, SBF was renewed to avoid saturation of the medium. The Ni released was quantified by Graphite Furnace Atomic Absorption Spectroscopy (GFAAS) (Ewai, Beijing, China) at 1 and 5 h, and 1, 2, 5, 15 and 30 days. The results were normalized by the specific surface area of each sample and are mean values of three measurements.

For the *in vitro* studies, human osteoblast-like MG63 cells (American Type Culture Collection, Rockville, MD, USA) were grown in Dulbecco’s modified Eagle’s medium (DMEM, Gibco, Paisley, UK) supplemented with 10% fetal calf serum (FCS), 1% penicillin/streptomycin, 1% L-glutamine, 1% pyruvate (Sigma, St. Louis, MO, USA) at 37 °C in 5% CO_2_/95% air atmosphere and 100% humidity. For the experiments, cells were harvested at 70%–90% confluence by Tripsin/EDTA, centrifuged and re-suspended in serum-free medium before plating on the samples. Samples were placed into a 24-well tissue culture plates (TCPS) (Falcon, Becton Dickinson & Company, Madrid, Spain) then 3 × 10^4^ cells per sample were added and incubated for 2 h in serum free medium. To observe cell adhesion and the overall morphology of adhering cells, the samples were stained with fluorescein diacetate (FDA) added directly to the medium to give a final concentration of 1 μg/mL (from a stock of 1 mg/mL in acetone) for 3 min. The stained living cells, the only ones able to convert the die to a fluorescent analog, were viewed and photographed with an inverted fluorescent microscope (Nikon, Eclipse E600, Barcelona, Spain) using the green channel.

For the *in vivo* studies, twelve female adult New Zealand White (NZW) rabbits (3.5 kg b.w., Charles River, Saint Aubin les Elboeuf, France) were operated on under general anesthesia, performed by intramuscular injections of xylazine (5 mg/kg) and ketamine (35 mg/kg). After lateral bilateral knee arthrotomy, a drilled bone defect of 4 mm in diameter and 6 mm deep was centered on the lateral condyle. All animal handling and surgical procedures were conducted according to European Community guidelines for the care and use of laboratory animals (DE 86/609/CEE) and approved by the local veterinary school ethical committee.

For the histological analysis, femoral condyles were harvested and peripheral soft tissue was removed. Specimens were fixed for 7 days in 4% formaldehyde neutral solution rinsed in water, dehydrated in graded series of ethanol (from 70% to 100%) and embedded in polymethyl methacrylate. The percentage of bone implant contact (BIC) was calculated using a semi-automatic binary treatment on each image. Qualitative examinations were performed by light microscopy on stained sections (1% methylenblue and 0.3% basic fuchsin).

For statistical analysis, all data were analyzed by a nonparametric U Mann-Whitney test (IBM SPSS Statistics 20 software, Armonk, NY, USA). Statistical significance was set at a *P* value < 0.05.

## 3. Experimental Results

Depth profiling of the O_2_-oxidized Ti was performed by curve fitting of the Ti 2p spectrum, which gave an estimation of the contribution of the TiO_2_ oxide in the total titanium signal (Table 1). Ti 2p spectrum was deconvoluted into eight peaks corresponding to four chemical states from doublet consisting of Ti 2p_1/2_ and Ti 2p_3/2_ (spectra not shown): two strong Ti^4+^ peaks, *i.e.*, 2p_1/2_ (Ti^4+^, 464.6 eV) and 2p_3/2_ oxide (Ti^4+^, 458.8 eV), two Ti^3+^ peaks (457 and 463.1 eV) and two Ti^2+^ peaks (455.3 and 461.7 eV), as well as two metallic Ti peaks (454.3 and 460.5 eV). The high-resolution Ti 2p XPS spectra were comparable to those obtained by other authors [22,23].

**Figure 1:**
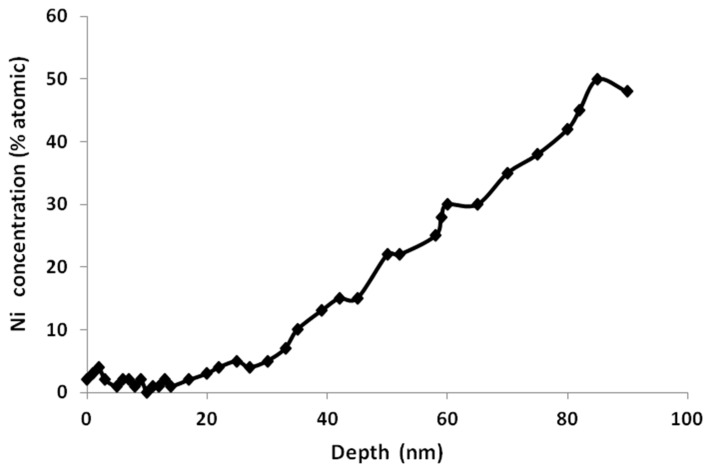
XPS depth profile of the nickel for austenitic alloy, after oxidation treatment.

For untreated samples, the Ti 2p photopeaks obtained indicate the presence of a stoichiometric TiO_2_, as well as Ti^0^ signals indicating that protective oxide layer thickness is very thin (below the depth limit of XPS). However, for the oxidized samples, it can clearly be observed that Ti is mostly present as Ti^4+^ and the Ti^0^ signal is practically negligible showing that the oxide layer has grown. Depth profiling reveals that the Ti^+4^ and Ti^+3^ species comprise the outermost of the oxide layer; the remaining oxide layer, close to the metal–oxide interface, consists of Ti^+2^ and Ti^0^ Finally, the depth profiling of the Ni as a function of sputtering time is plotted in Figure 1, where a zone depleted in Ni (<3 at %) is formed up to approximately 25 nm in depth. It is expected that the presence of a relatively thicker TiO_2_ layer, free of nickel, could prevents the nickel ions release to the surrounding tissues.

**Figure 2:**
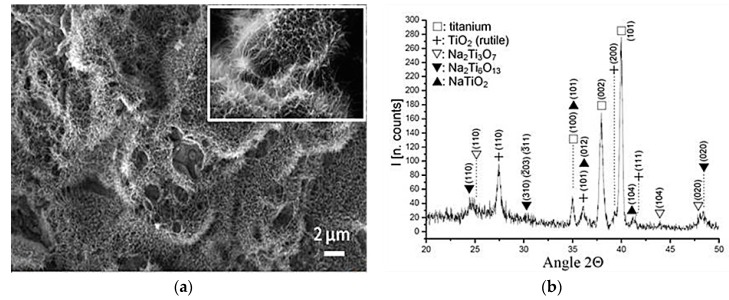
(**a**) SEM micrograph showing a microporous layer of sodium titanate on NiTi surface (alloy A); and (**b**) GI-XRD pattern of sodium titanate layer.

**Figure 3:**
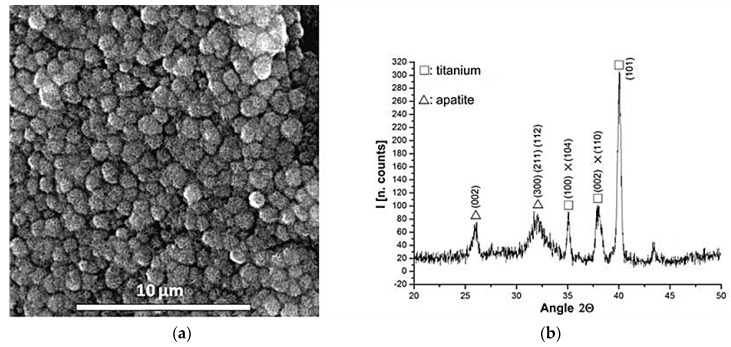
(**a**) SEM micrograph of apatite layer on NiTi surface (Alloy A) after soaked in SBF for three days; and (**b**) GI-XRD pattern showing peaks corresponding to the apatite crystals (32° and 26°).

Figure 2 shows the appearance of the samples treated with NaOH solution and heated up to 600 °C. A microporous layer made up of an alkaline titanate has been induced on the NiTi surface. The morphology obtained is very similar to other studies of biomimetic surfaces treatments performed on titanium [24]. The corresponding GI-XRD patterns are very similar for both NiTi alloys (austentic and martensitic) showing peaks corresponding to metallic nickel-titanium, rutile and different sodium-titanates stoichiometries, for example, Na_2_Ti_3_O_7_ (JCPDS #72-0148), Na_2_Ti_6_O_13_ (JCPDS #73-1398) and NaTiO_2_ (JCPDS #89-2784). Finally, when the alkaline titanate hydrogel surfaces were immersed in SBF at 37 °C for a period of time as short as three days, an apatite layer appeared (Figure 3). The morphology obtained was consistent with those reported in the literature [25] and the nature of this bioactive layer was confirmed by GI-X-ray diffractometer. It showed peaks corresponding to apatite crystals with almost non-existent alkaline titanates peaks (Figure 3a). The XRD peaks for apatite near 32° and 26° are evident.

Ni ions release concentration as a function of immersion time in SBF at 37 °C was evaluated. The experimental results showed that the Ni ion release is efficiently reduced and the highest Ni release occurs within the first hour. After 24 h of immersion, it was negligible. The total Ni release after one month of immersion was about 100 ± 32, 32 ± 5, and 10 ± 4 ng/cm^2^ for control, oxidized, and bioactive surfaces, respectively. This corresponds to respective mean values of 0.29, 0.13 and 0.04 μg/mL in our system.

**Figure 4:**
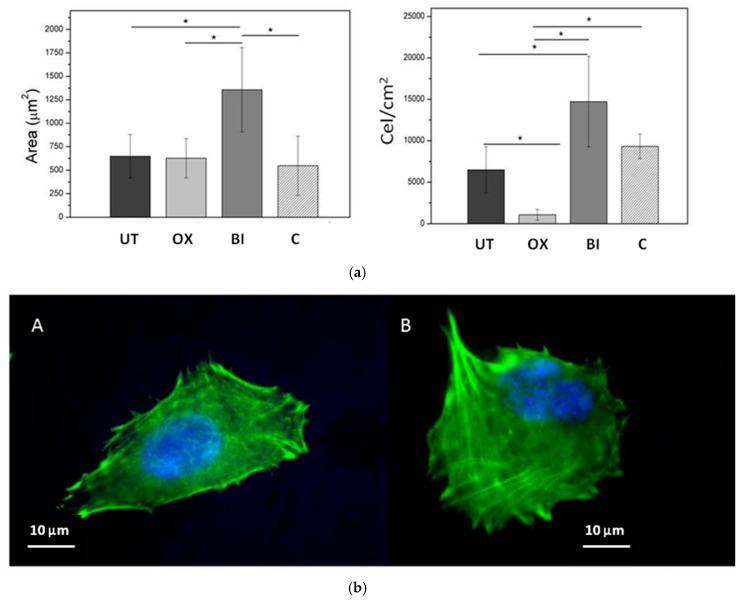
(**a**) Cell spreading and cell number after 2 h of culture corresponding to different surfaces: **C**, Teflon; **UT**, untreated Ti; **OX**, oxidized Ti; and **BI**, bioactive Ti; (**b**) Representative fluorescence microscopy images of MG63 cells on untreated NiTi (**A**) and bioactive NiTi (**B**) after seven days of incubation (alloy A).

The surface modification proposed induced higher cell recruitment and cell spreading after only 2 h of culture (Figure 4a). Similarly, cell spreading quantification presented considerably higher area values for the bioactive NiTi (1350 ± 240 μm^2^), and no differences were observed among the other samples. Immunofluorescence images showed that cells on untreated NiTi surface exhibited a flattened and less-extended morphology spread than those observed in the bioactive NiTi (Figure 4b). Large cytoplasmic extensions like filopodia anchored the cell to the substrate and dorsal activity have been observed in the osteoblast cultured on the bioactive surface in relation to others samples.

**Figure 5:**
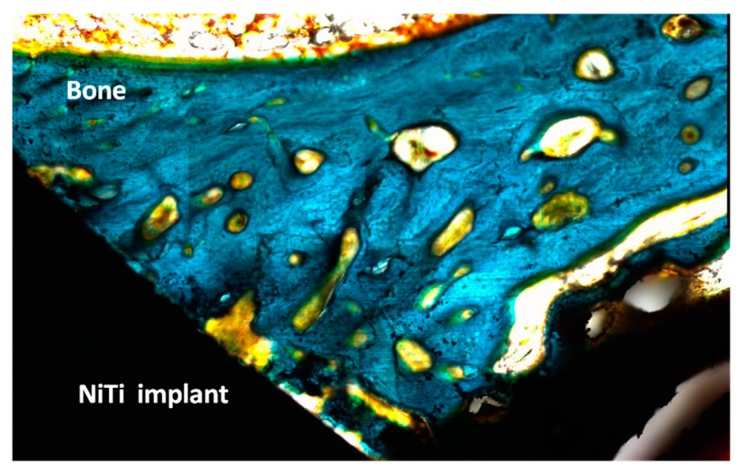
Histological micrograph showing the interface bone–implant for the bioactive NiTi alloy (×400). The implant–bone interface is almost completely covered after six weeks of implantation.

Finally, Table 2 summarizes the percentage of the bone implant contact (BIC) at microscopic level for untreated and bioactive implants, respectively. The control NiTi implants had lower percentages of direct contact bone with statistically significant differences (Fisher’s test; 0.05) than the thermochemical treated samples. After one week, BIC values were significantly higher for the bioactive implants, ranging between 22% and 32%, while the mean values for the untreated NiTi implants ranged between 5% and 13%. This means that an osseointegration is achieved for bioactive implants at times of implantation as brief as 1 week. Overall, the specimens showed the presence of remodeling activity in the bone immediately adjacent to the microimplant. The newly-formed bone showed early stages of maturing and remodeling, particularly on the treated surface (Figure 5).

Finally, Table 3 shows the transformation temperatures (A_s_, A_f_, M_s_, and M_f_) for untreated and bioactives NiTi alloys. The DSC curves at different cooling/heating cycles do not shown differences and R-phase peaks were not observed. For both microstructures (austenite/martensite), a decrease of the transformation temperatures was observed due to the thermochemical treatment performed. Moreover, the critical stresses required to induce the stress-induced martensite, σ^β→M^ or σ^M→β^ at 20 °C and 37 °C, respectively, have been changed. The transformation stresses for the autenitic NiTi alloy are shown in Table 4. It is clearly shown that higher stresses were required to transform the austenite to stress-induced martensite and *vice versa* in the treated samples.

## 4. Discussion

In the first step, a thicker protective layer of rutile/anastase, free of nickel, has been produced on the NiTi surfaces. The oxidation process at low oxygen pressure allowed a decrease of Ni concentration on the surface (Figure 1). In the literature, studies of NiTi oxidation with similar results exist [22,23,26,27]. For example, our findings are similar to those obtained by Chan *et al.* [28] using an oxidation treatment. Surface oxidation produced a smooth protective nickel-free oxide layer with a relatively small amount of Ni species at the air/oxide interface. Studies published in the literature could be established to explain these results [20]. According to Armitage *et al.* [29], the oxide layer is predominantly formed by inward diffusion of O ions inside NiTi lattice. Because ΔG_formation (298K)_ for TiO_2_ and NiO are −212.6 kcal∙mol^−1^ and −50.6 kcal∙mol^−1^, respectively [30,31], there is a preferential oxidation of Ti. Ni remains in its metallic state. The formation of stoichiometric TiO_2_ is progressive and TiO*_x_* (*x* < 4) may be present underneath [23] at the TiO_2_-NiTi interface. In that situation, Espinós *et al.* [32] showed that Ni atoms in metallic state diffuse easily through defective TiO*_x_* (*x* < 4). There is a migration of Ni atoms leading to its disappearance from the surface. This could explain the Ni-enriched layer observed below the Ni-depleted zone in the concentration profiles obtained.

In the second step, the formation of a sodium alkaline titanate hydrogel from oxidized surface has been induced (Figure 2). It is reported that the apatite forming ability is dependent on the titania crystalline structure. The titania gels with amorphous structure did not induce apatite formation on their surfaces in SBF, whereas titania with anastase/rutile structure induced apatite formation [33]. In the present study, GI-XRD studies have shown a crystallographic layer composed by rutile as well as different sodium titanates crystals (Figure 2). Moreover, the simulated body fluid (SBF) corroborated the ability to induce apatite formation (Figure 3). It is well known that the SBF, which has a similar ion concentrations as those of the human blood plasma, could reproduce *in vivo* surface changes [34]. The good results obtained before (Figure 3) are corroborated *in vitro* and *in vivo* cells studies. The bioactivity of the NiTi modified surface is improved (Figure 4 and Figure 5). Bone implant contact values indicated that the osseointegration process is improved (Table 3).

The release of Ni ions can be harmful to the cells of the adjacent tissues and it is closely linked to the corrosion behavior. They are important parameters to take into account for medical devices as they can have long-term *in vivo* implications [7,8]. There are studies showing that Ni ion release is able to induce allergic response [35,36,37,38]. For example, Cederbrant *et al.* [39] showed that even a quantity as small as 1.2 μg/mL of Ni could induce an increase in lymphocytes proliferation and IL10 secretion in subjects allergic to Ni. Thus, the results obtained for our bioactive surfaces (0.05 μg/mL) might be of great importance to improve the long-term biocompatibility properties.

Numerous studies on the development of new biomimetic surfaces on NiTi alloys are reported; however, they do not pay attention to how the pseudoelastic or shape memory properties could be affected by these treatments. For example, Table 3 clearly shows that higher stresses were required to transform the austenite to stress-induced martensite and *vice versa* in the treated samples. These higher stresses are, probably, due to the transformation temperature (M_s_) was far from 37 °C for treated samples (Table 3). It is not clear why the transformation temperatures (A_s_, A_f_, M_s_, and M_f_) decrease due to the thermo-chemical treatment. One hypothesis could be the formation of the titanium-rich precipitates into the NiTi bulk decreasing the titanium content during the thermochemical treatment. For example, Arciniegas *et al.* [37] have demonstrated the appearance of NiTi precipitates rich in Titanium (Ti_2_Ni) when a NiTi alloy (50 at % with martensitic microstructure) are subjected at similar heat treatments. Ti_2_Ni precipitates impeded the growth of martensitic plates and can be nucleation source of them. Similar observations were reported by Firstov [23] where the martensitic transformation of the substrate NiTi alloy beneath the oxidized scale is suppressed below room temperature because of the depletion of the NiTi lattice by Ti which is consumed in the oxidized scale to produce Ti oxides. However, further studies by TEM will be carried out in the future.

Higher stress was needed to transform the austenite to stress-induced martensite in the bioactive samples because the transformation temperature was far from 37 °C than in the original samples. In terms of clinical applications, this small increase of the critical stresses (approximately 18 MPa) implies that higher forces will be applied to the bone tissues. The elastic deformation of a NiTi sample and the subsequent release of its elastic energy over a period of time increase the loads on the living tissue. It is generally assumed that optimal load transfer in the living tissues is achieved by applying forces that are low in magnitude and continuous in nature [40,41]. Fortunately, the thermochemical method proposed does not produce large variations, but this is a fact that must be taken into account in terms of clinical applications.

## 5. Conclusions

A new thermo-chemical treatment for inducing bioactivity on several NiTi alloys has been proposed. In the first step, a safe barrier against Ni release has been produced on the surface by means of a thicker rutile/anastase protective layer free of nickel. In the second step, a sodium alkaline titanate hydrogel, which has the ability to induce apatite formation, has been performed from oxidized surface. The bioactivity of the treated NiTi alloys has been corroborated with both *in vitro* and *in vivo* studies. Moreover, the improvement of the host tissue–implant integration in terms of Ni ions release and corrosion resistance has been demonstrated. However, the transformation temperatures (A_s_, A_f_, M_s_, and M_f_), as well as the critical stresses (σ^β⇔M^), have been slightly modified due to the surface modification. In terms of clinical applications, the variation of the critical stresses implies that higher or lower forces will be applied to the bone tissues.

## Figures and Tables

**Table 1:** Variation of the titanium oxide composition as a function of depth for different surface treatments studied (UT: untreated samples; OT: treated samples).

Treatment	Sputtering Time (min)/Depth (nm)	Chemical Element	% of total Ti 2p Signal
UT	0/0	Ti^0^ Ti^+2^, Ti^+3^/Ti^+4^	8/10/82
OT	0/0	Ti^+4^	100
OT	1/6	Ti^3+^ Ti^4+^	61/39

**Table 2:** Bone index contact after one and six weeks of implantation (columns) in percent.

Type of Implants	1 Week	6 Weeks
NiTi	9% (±4%)	44% (±11%)
NiTi bioactive	27% (±5%)	68% (±15%)

**Table 3:** Transformation temperatures (°C) for the Ni-Ti studied.

Alloy	M_s_	M_f_	A_s_	A_f_
M	74.1 ± 0.3	55.7 ± 0.4	86.9 ± 0.1	129.3 ± 0.7
M-bioactive	64.2 ± 0.6	46.1 ± 0.5	71.0 ± 0.1	109.3 ± 0.7
A	9.8 ± 0.2	6.2 ± 0.3	7.1 ± 0.4	20.4 ± 0.5
A-bioactive	0.5 ± 2.4	−3.9 ± 1.2	−0.4 ± 1.7	11.2 ± 4.5

**Table 4:** Critical stresses at different test temperatures for austenitic NiTi alloy.

Autenitic NiTi Alloy	Critical Stresses	20 °C	37 °C
NiTi original	σ^β→SIM^ (MPa)	150 ± 17	262 ± 24
	σ^SIM→β^ (MPa)	55 ± 5	211 ± 19
NiTi bioactive	σ^β→SIM^ (MPa)	180 ± 15	290 ± 22
	σ^SIM→β^ (MPa)	65 ± 9	183 ± 19
